# Pregnancy Risk Assessment Monitoring System for Dads: A piloted randomized trial of public health surveillance of recent fathers’ behaviors before and after infant birth

**DOI:** 10.1371/journal.pone.0262366

**Published:** 2022-01-21

**Authors:** Craig F. Garfield, Clarissa D. Simon, Fay Stephens, Patricia Castro Román, Michael Bryan, Ruben A. Smith, Katherine Kortsmit, Beatriz Salvesen von Essen, Letitia Williams, Martha Kapaya, Ada Dieke, Wanda Barfield, Lee Warner

**Affiliations:** 1 Department of Pediatrics, Northwestern University Feinberg School of Medicine, Chicago, Illinois, United States of America; 2 Division of Hospital Medicine, Ann & Robert H. Lurie Children’s Hospital of Chicago and Stanley Manne Children’s Research Institute, Chicago, Illinois, United States of America; 3 Family and Child Health Innovations Program, Ann & Robert H. Lurie Children’s Hospital of Chicago and Stanley Manne Children’s Research Institute, Chicago, Illinois, United States of America; 4 Georgia Department of Public Health, Department of Epidemiology, Atlanta, Georgia, United States of America; 5 Georgia Department of Public Health, Department of Maternal and Child Health Epidemiology, Atlanta, Georgia, United States of America; 6 Centers for Disease Control and Prevention, Division of Reproductive Health, Atlanta, Georgia, United States of America; Montclair State University, UNITED STATES

## Abstract

**Background:**

Becoming a father impacts men’s health and wellbeing, while also contributing to the health and wellbeing of mothers and children. There is no large-scale, public health surveillance system aimed at understanding the health and behaviors of men transitioning into fatherhood. The purpose of this study was to describe piloted randomized approaches of a state-based surveillance system examining paternal behaviors before and after their infant’s birth to better understand the health needs of men and their families during the transition to parenthood.

**Methods:**

During October 2018–July 2019, 857 fathers in Georgia were sampled 2–6 months after their infant’s birth from birth certificates files and surveyed via mail, online or telephone, in English or Spanish, using two randomized approaches: Indirect-to-Dads and Direct-to-Dads. Survey topics included mental and physical health, healthcare, substance use, and contraceptive use.

**Findings:**

Weighted response rates (Indirect-to-Dads, 33%; Direct-to-Dads, 31%) and population demographics did not differ by approach. Respondents completed the survey by mail (58%), online (28%) or telephone (14%). Among 266 fathers completing the survey, 55% had a primary care physician, and 49% attended a healthcare visit for themselves during their infant’s mother’s pregnancy or since their infant’s birth. Most fathers were overweight or had obesity (70%) while fewer reported smoking cigarettes (19%), binge drinking (13%) or depressive symptoms (10%) since their infant’s birth.

**Conclusions:**

This study tests a novel approach for obtaining population-based estimates of fathers’ perinatal health behaviors, with comparable response rates from two pragmatic approaches. The pilot study results quantify a number of public health needs related to fathers’ health and healthcare access.

## Introduction

Fathers represent nearly two-thirds of the adult male population in the United States [1] and can play key roles in the health and development of their families [2]. Father involvement has been linked to improved maternal and infant health, including longer breastfeeding duration [3], lower levels of maternal depression [4], earlier prenatal care initiation [5], higher utilization of postnatal care services [6], and improved child developmental, psychological and cognitive outcomes [7, 8]. Beyond influencing the health of their families, fatherhood presents an opportunity for men to improve their own health [9]. Healthy men are more likely to participate in childrearing [10], support mothers in parenting [11], and have healthy children [12].

Specifically including fathers in health studies over the transition to parenthood is an increasingly recognized need [13]; however, no population-based surveillance system in the United States currently collects data from fathers during the perinatal period [14]. While several federally-funded, population-based surveys include men (e.g., National Health Interview Survey [NHIS] [15], Behavioral Risk Factor Surveillance System [BRFSS] [16], National Survey of Family Growth [NSFG] [17], and National Health and Nutrition Examination Survey [NHANES] [18]), they are not specific to fatherhood or men’s experiences and behaviors before, during, and after the perinatal period. Further, while there are several father-inclusive population-based studies (e.g., Fragile Families and Child Wellbeing Study [4], Early Childhood Longitudinal Study of Birth [ECLS-B]) [19], they do not specifically focus on the health of fathers during the perinatal period.

Since 1987, the Pregnancy Risk Assessment Monitoring System (PRAMS), a site-specific and population-based surveillance system, has collected data annually on a representative sample of mothers regarding their experiences, attitudes, and behaviors before, during, and shortly after pregnancy from births sampled 2–6 months after delivery, with most respondents answering the survey at 3–4 months postpartum [20]. Building on the PRAMS infrastructure, a parallel population-based survey for fathers during the perinatal period, known as “PRAMS for Dads,” was implemented in collaboration with the Georgias Department of Public Health [2]. The purpose of the study was to 1) test two methodological approaches for effectively reaching a representative sample of fathers of recent live-born infants, and 2) describe fathers’ behaviors and experiences during the perinatal period, including before and shortly after the birth of their infant.

## Materials and methods

We conducted a pilot, randomized controlled trial comparing response rates for two methodological approaches for sampling fathers 2–6 months following the birth of an infant. Eligibility criteria included Georgia residency, in-state birth, and presence of both maternal and paternal identifying information on the birth certificate. Unmarried fathers without a paternity acknowledgement (PA) form (N = 14,887), and fathers with unknown identifying information, including marital status or presence of a PA (N = 65) were excluded from eligibility from the study due to the target population definition. The target population was comprised of fathers in Georgia who were listed directly on the birth certificate through marriage or listed indirectly via a completed PA form [21]. The study protocol, modeled after PRAMS [20], used stratified random sampling to select women with a recent live birth from birth certificate files and was designed to reflect Georgia’s birth population. To align with ongoing PRAMS operations in Georgia, we selected all eligible fathers (hereafter “sampled fathers”) for whom the infant’s mother had been sampled in PRAMS during the period from October 15, 2018–July 3, 2019 (representing infants born during May 28, 2018–May 3, 2019). During the study period Georgia PRAMS sampled 1,074 women with a live birth, eligible fathers could be identified for 857 (79.8%) births and were included in the "PRAMS for Dads" pilot. This study was approved by both the Georgia Department of Public Health Institutional Review Board, which served as the Institutional Review Board of record, and the Northwestern University Institutional Review Board.

Following the PRAMS data collection protocol for mothers [20], sampled fathers were mailed a pre-survey letter describing the study, followed by the first survey packet, a reminder letter, and two additional survey packets. Survey packets included a cover letter, survey instrument, pre-stamped return envelope, flat pen with the PRAMS logo (provided as a gift to encourage response), and a resource list including support services for a variety of needs from child support and career services to fatherhood and parenting (e.g., Department of Human Services—Division of Child Support Services, Georgia Department of Labor Career Centers, Georgia Fatherhood Program). Mail non-respondents were followed up with telephone outreach beginning approximately 45 days after initial contact per PRAMS protocol, continuing with up to 15 call attempts made until the index infant was 9 months old. Fathers who completed the survey received a $20 gift card, the same gift offered to mothers participating in Georgia PRAMS.

To assess the most effective way to reach fathers, we randomized fathers to one of two study arms: 1) “Indirect-to-Dads,” wherein fathers were contacted indirectly through the infants’ mother, or 2) “Direct-to-Dads,” where fathers were contacted directly. The Indirect-to-Dads arm borrows from existing literature referring to mothers as gatekeepers or gateopeners, conveying the idea that mothers can either block or facilitate access to fathers [22, 23]. This indirect method of reaching fathers is a validated approach in other studies surveying fathers in the perinatal period [24]. Participants in the Indirect-to-Dads arm were therefore contacted using the available mailing address for mothers from the birth certificate, and materials for fathers were included in the same mailing packet sent to mothers selected for participation in the Georgia PRAMS survey. All materials were addressed to the sampled PRAMS mother, rather than the father, who was then asked to provide the PRAMS for Dads survey to the infant’s father to complete. When fathers did not respond to the mailings, mothers were subsequently contacted via telephone and asked to provide the father’s contact information. No further attempts to contact fathers were made when contact information was not provided by the infant’s mother. Participants in the Direct-to-Dads arm were contacted using the fathers’ physical (residential) address listed on the birth certificate since mailing address was not reported for the second parent; all mailings were addressed and sent directly to fathers, independent of the mother. Fathers who did not respond to the mailings were directly contacted via telephone numbers first obtained through the following external database sources: LexisNexis, Thomson Reuters CLEAR investigation software, and Georgia Registry of Immunization Transactions & Services, and Newborn Screening databases. If external databases were unable to find a father’s phone number, mothers’ phone numbers available on the birth certificate were contacted to reach fathers. Mothers were then asked to provide father’s contact information, including during her PRAMS phone interview, if she had not yet responded by mail. A third option for survey participation, web-based completion, which is not currently available for regular PRAMS, was made available to all fathers via a personal link included in the preletter and all subsequent mailings, regardless of study arm.

The PRAMS for Dads survey instrument, modeled after the PRAMS questionnaire for mothers (https://www.cdc.gov/prams/questionnaire.htm), was available in Spanish and English, took approximately 30 minutes to complete, and contained 71 questions covering a range of topics including health care access and usage, contraceptive use, health behaviors, infant safe sleep practices, father involvement, and employment. Survey questions were directly taken or adapted from validated surveys [17, 20, 25].

The number of contact attempts and survey completions for participants in both arms was monitored for mail, telephone, and web options by Georgia Department of Public Health (GDPH) study staff using Research Electronic Data Capture (REDCap), a secure web-based data collection and management platform [26]. All mail surveys were double-entered and verified independently by two study staff members.

Survey data were weighted for sampling design, nonresponse, and noncoverage to be representative of fathers to live-born infants who were either married or unmarried, with a completed PA form, in Georgia. The noncoverage weight accounts for fathers from the target population that were excluded from the selection process of the sample because their names were not listed on the birth certificate; the percentage of noncoverage was 1.6%, representing the total number of fathers with missing last name (1,336) divided by the total number of fathers in the target population (85,653). Paternal characteristics and infant birth outcome data, collected from infant birth certificates, included age, education, race/ethnicity, indication of paternity (either married or unmarried with signed PA form), infant gestational age at delivery and infant birthweight. Paternal body mass index (BMI) was computed using self-reported weight and height values survey responses. The two-item patient health questionnaire (PHQ-2) was used to determine frequency of depressed mood after the infant’s birth.

We used Chi-squared tests to compare the distribution of select paternal characteristics by study arm among sampled fathers, and used Chi-squared testing, followed by Benjamini and Hochberg [27] adjusted Chi-squared testing for post-hoc analyses involving variables with more than two categories in cases of a significant Chi-squared test, to compare the distribution of study arm, paternal characteristics and infant birth outcomes between respondents and non-respondents. Finally, we calculated weighted prevalence estimates and 95% confidence intervals of select demographic and health-related survey measures among respondent fathers. Data analyses were conducted by two co-authors who were blinded to study arm allocation to prevent detection and interpretation bias; this was achieved by labeling as arm 1 and 2 during the analytic process.

All analyses were conducted in SAS-callable SUDAAN v. 11.0.1 (RTI International, Research Triangle Park, NC, USA) to account for the complex survey design. The statistical significance level was set at p-value <0.05. Power and sample size calculations indicated that a minimum of 388 fathers would need to be sampled for each arm (776 in total) to detect a difference of at least 10% in response rates at 80% power and 5% level of significance (2-sided) [28].

Verbal/oral consent was obtained during phone phase using a consent script prior to completing the survey over the phone. Voluntarily returning the survey via mail, completing the survey electronically, or completing the telephone interview was considered a tacit indication of participant consent. The Centers for Disease Control and Prevention was not directly engaged in the research, and did not interact with study participants or have access to identifiable information pertaining to the fathers.

## Results

### Study sample

Of 857 fathers comprising the study sample, 429 were randomized to the Indirect-to-Dads arm and 428 to the Direct-to-Dads arm. Compared with all fathers of live-born infants in Georgia during the study period who were either married or unmarried with a PA form (N = 85,653), weighted survey respondents for PRAMS for Dads did not differ significantly on selected paternal characteristics, including age, education, race/Hispanic origin, and indication of paternity (Table 1). Overall, 266 fathers (31.7% weighted response rate) completed the survey; of these, 132 were in the Direct-to-Dads arm and 134 were in the Indirect-to-Dads arm (Table 1). The weighted proportion of respondent fathers who were married was 64.6%, with 55.7% completing at least some college, 37.7% aged 35 or older, and 48.3% of fathers were non-Hispanic white.

**Table 1:** Comparison of selected paternal characteristics of the PRAMS for Dads pilot study target population and weighted survey respondents, October 2018–July 2019, Georgia, USA.

Selected paternal characteristics from the birth certificate	PRAMS for Dads target population ^a^		Weighted survey respondents		
	N = 85,653^b^		n = 266^c^		
	N	%^f^	n	%^g^	P-Value^d^
**Age, years**					
<25	13,520	16	31	12.6	0.4438
25–34	40,841	48.3	129	49.7	
> = 35	30,181	35.7	106	37.7	
**Education**					
≤High school or GED	40,705	48.6	123	44.3	0.5079
Some college, no degree	13,523	16.1	47	19.3	
College Grad	29,609	35.3	96	36.4	
**Race/Hispanic origin**					
Non-Hispanic white	37,759	45.4	121	48.3	0.6944
Non-Hispanic black	27,044	32.5	73	30.8	
Hispanic	12,216	14.7	50	12.6	
Non-Hispanic other/Multiple Races^e^	6,241	7.5	19	8.3	
**Indication of Paternity**					
Married	54,674	63.8	193	64.6	0.8469
Unmarried with PA form	30,979	36.2	73	35.4	

Abbreviations: PA = Paternity Acknowledgement, GED = General Educational Development.

^a^ The taret population for the Georgia PRAMS for Dads pilot study is all Georgia residents who are fathers to a live-born infant. Only married fathers or unmarried fathers with completed paternity acknowledgment form were included in the population of interest. Exclusions: Because the definition of the target population, certain fathers were excluded from eligibility in the Georgia PRAMS for Dads survey: Unmarried fathers without paternity acknowledgment forms or unknown marital status. Study period includes infants born between May 28, 2018 to May 3, 2019.

^b^ Totals may not add up to the indicated PRAMS for Dads target population size (N = 85,653) due to missing/unknown values.

^c^ Totals may not add up to the indicated overall unweighted sample size (n = 266) due to missing/unknown values.

^d^ Goodness-of-fit (GOF) p-value. GOF hypothesis: Distributions of the PRAMS for Dads target population and weighted sample of survey respondent percentages are statistically equivalent.

^e^ Race categories API (n = 14) and Other/Multiple races (n = 5) were combined because of small sample size.

^f^ Weighted percentage.

^g^ Father analysis weight is the product of his sampling weight, non-response weight and non-coverage weight.

### Impact of methodology on response

**Fig 1:**
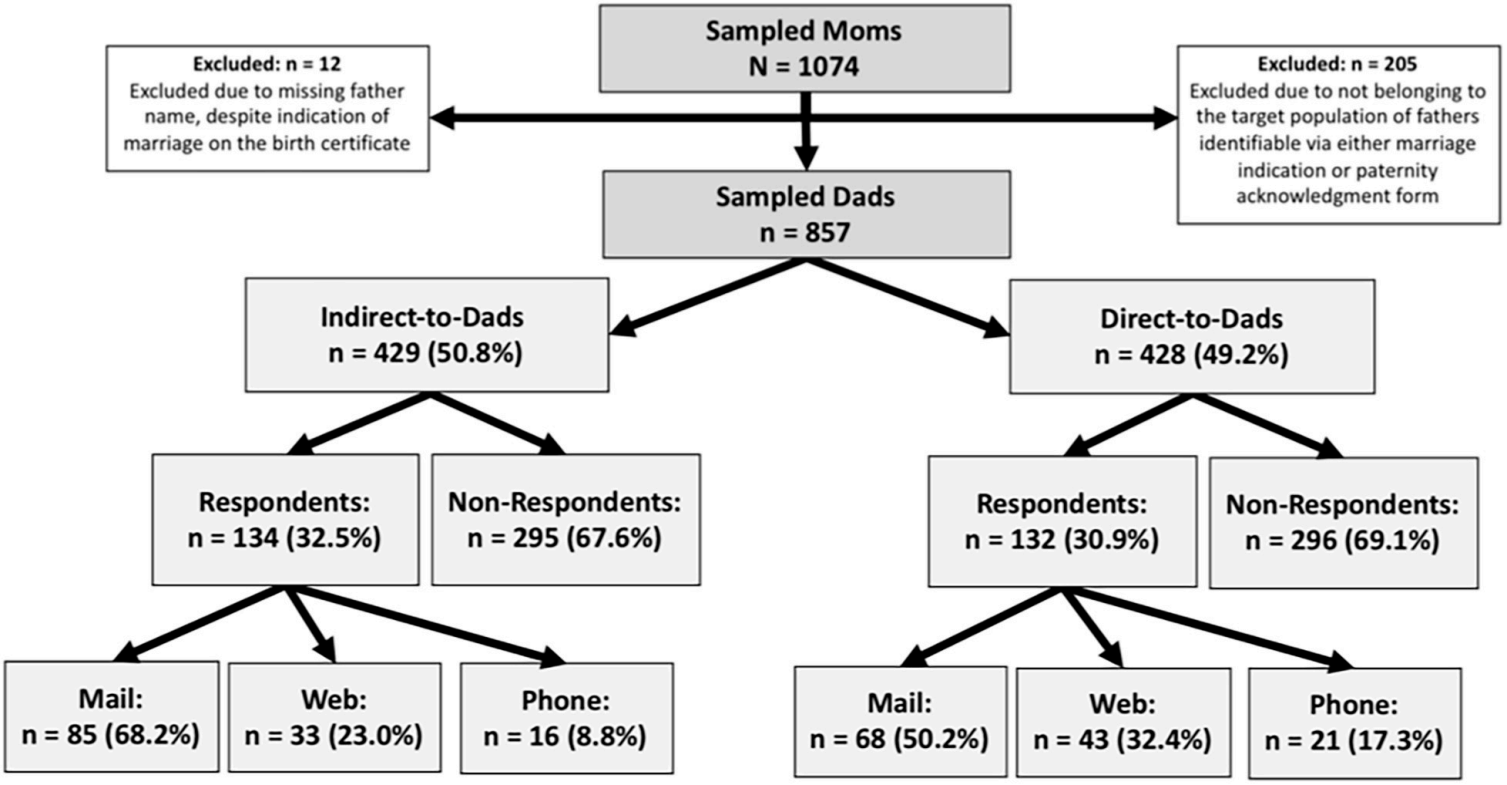
Flow chart of the selection of eligible participants sampled the Pregnancy Risk Assessment Monitoring System for Dads Pilot Study by study arm, survey response, and mode of survey completion. NOTE: All percentages are weighted. Father analysis weight is his sampling weight. Numbers may not sum to 100% due to rounding.

Overall response rates did not differ by study arm (Fig 1: 32.5%, Indirect-to-Dads; 30.9%, Direct-to-Dads), The majority of fathers in both arms responded to the survey by mail (68.2%, Indirect-to-Dads, 50.2%, Direct-to-Dads), followed by online (23.0%, Indirect-to-Dads; 32.4%, Direct-to-Dads) and phone (8.8%, Indirect-to-Dads; 17.3%, Direct-to-Dads). Paternal age, education, race and Hispanic origin, marital status, education, infant gestational age at birth and infant birthweight, as obtained from the birth certificate file, were distributed similarly between the two study arms among sampled fathers (Table 2). When comparing respondents and non-respondents, no significant differences were observed by study arm, paternal age, paternal education, infant gestational age at birth, or infant birthweight (Table 3). Compared with non-respondents, a higher proportion of respondents were Hispanic (10.8% vs 19.5%), and a lower proportion of respondents were non-Hispanic Black (39.7% vs 25.6%) and unmarried with a completed PA form (42.7% vs 26.7%).

**Table 2:** Distribution of select characteristics from the birth certificate: Comparison between study arms^a^—Total sampled fathers–the Pregnancy Risk Assessment Monitoring System for Dads study, October 2018-July 2019, Georgia, USA.

	Direct-to-Dads	Indirect-to-Dads
	n = 428^b^	n = 429^a^^b^
Characteristics from the birth certificate	n^b^ (%^c^)	n^b^ (%^c^)
***Paternal Demographic Characteristics***		
**Age**		
<25	68 (16.9)	72 (15.3)
25–34	213 (48.5)	197 (46.9)
≥35	147 (34.5)	159 (37.8)
**Education**		
≤High school or GED	231 (50.8)	208 (47.3)
Some college, no degree	78 (20.7)	83 (17.6)
Bachelor’s degree or higher	119 (28.5)	135 (35.1)
**Race/Hispanic origin**		
Non-Hispanic white	184 (46.7)	175 (42.3)
Non-Hispanic black	159 (35.8)	161 (34.5)
Hispanic	54 (11.9)	60 (15.2)
Non-Hispanic other	23 (5.6)	26 (7.9)
**Indication of Paternity**		
Married	261 (61.7)	258 (63.0)
Unmarried with PA form	167 (38.3)	171 (37.0)
***Infant Birth Outcomes***		
**Infant gestational age at birth (weeks)**		
Preterm, <37	206 (10.2)	199 (9.5)
Term, ≥37	222 (89.8)	230 (90.5)
**Infant birthweight (grams)**		
Low birthweight, <2500	118 (8.0)	115 (7.2)
Normal birthweight, ≥2500	310 (92.0)	314 (92.8)

Abbreviations: PA = Paternity Acknowledgement, GED = General Educational Development.

^a^Distribution of select paternal characteristics comparisons between study arms are made using a Chi-squared test at the significance level of 0.05. All comparisons exceed the significance level.

^b^ Unweighted sample size; sample size may vary due to missing.

^c^ Weighted percentage. Father analysis weight is his sampling weight.

**Table 3:** Distribution of study arm and select characteristics by survey participation—The Pregnancy Risk Assessment Monitoring System for Dads study, October 2018–July 2019, Georgia, USA.

Study arm/Characteristics from the birth certificate	Sampled fathers (N = 857)	
	Respondents	Non-Respondents
	n^a^ (%^b^)	n^a^ (%^b^)
**Study Arm**		
Direct-to-Dads	132 (47.9)	296 (49.8)
Indirect-to-Dads	134 (52.1)	295 (50.2)
***Paternal Demographic Characteristics***		
**Age**		
<25	31 (12.4)	109 (17.8)
25–34	129 (50.0)	281 (46.7)
≥35	106 (37.6)	200 (35.5)
**Education**		
≤High school or GED	123 (45.0)	316 (50.9)
Some college, no degree	47 (17.0)	114 (20.2)
Bachelor’s degree or higher	96 (38.0)	158 (29.0)
**Race/Hispanic origin** ^c^		
Non-Hispanic white	121 (47.3)	238 (43.1)
Non-Hispanic black^d^	73 (25.6)	247 (39.7)
Hispanic^d^	50 (19.5)	64 (10.8)
Non-Hispanic other	19 (7.6)	30 (6.4)
**Paternity type** ^c^		
Married^c^	193 (73.3)	326 (57.3)
Unmarried with PA form^c^	73 (26.7)	265 (42.7)
***Infant Birth Outcomes***		
**Infant gestational age at birth (weeks)**		
Preterm, <37	122 (9.4)	283 (10.1)
Term, ≥37	144 (90.6)	308 (89.9)
**Infant birthweight (grams)**		
Low birthweight, <2500	60 (6.8)	173 (8.0)
Normal birthweight, ≥2500	206 (93.2)	418 (92.0)

Abbreviations: PA = Paternity Acknowledgement, GED = General Educational Development.

^a^ Unweighted sample size; sample size may vary due to missing.

^b^ Weighted percentage. Father analysis weight is his sampling weight.

^c^ Chi-squared test significant, p<0.05.

^d^ Benjamini and Hochberg-adjusted Chi-squared test significant, p<0.05 for difference between respondents and non-respondents (obtained by post-hoc analysis).

### Demographic and health characteristics of respondents

Most fathers (92.1%) reported working during their infant’s mother’s pregnancy (Table 4). Half (50.6%) reported a household income >200% of the federal poverty level, and nearly two-thirds (63.3%) reported having private insurance. Nearly half (48.6%) of respondents had attended any health care visit for themselves during their infant’s mother’s pregnancy or since their infant was born, while over half (54.8%) reported currently having a primary care physician. Among those attending any health care visit during their infant’s mother’s pregnancy or since the infant was born, 67.1% sought a regular checkup, 55.9% reported having their teeth cleaned, and 28.2% reported seeking care for an illness or chronic condition. Visits for an injury (8.0%) or family planning/birth control (8.2%) were less common.

**Table 4:** Prevalence of select demographic and health characteristics and behaviors among respondents—The Pregnancy Risk Assessment Monitoring System for Dads pilot study, October 2018–July 2019, Georgia, USA.

Select characteristics	n^a^	% (95% CI)^b^
**Worked for pay during infant mother’s pregnancy**	240	92.1 (86.3–95.5)
**Household income by federal poverty level (FPL)**		
0–100% FPL	46	21.3 (15.2–29.1)
101–200% FPL	70	28.1 (21.5–35.8)
>200% FPL	123	50.6 (42.5–58.7)
**Current insurance coverage**		
Private	160	63.3 (55.3–70.7)
Public	14^c^	6.2 (3.2–11.6)^c^
None	81	30.5 (23.6–38.4)
***Since Infant’s Mother’s Pregnancy or Since Infant’s Birth***		
**Attended any health care visit**	123	48.6 (40.8–56.4)
**Had a primary care physician**	134	54.8 (47.0–62.5)
**Type of healthcare visit** ^d^		
Regular checkup at your family doctor’s office	82	67.1 (55.6–76.9)
Visit for an illness or chronic condition	33	28.2 (19.0–39.7)
Visit for an injury	10^c^	8.0 (3.5–17.0)^c^
Visit for family planning or birth control	7^c^	8.2 (3.5–18.0)^c^
Visit for depression or anxiety	^e^	^e^
Visit to have teeth cleaned by a dentist or dental hygienist	62	55.9 (44.4–66.7)
**Body mass index**		
Underweight	10^c^	5.7 (2.8–11.4)^c^
Normal weight	59	24.5 (18.4–31.8)
Overweight	104	39.2 (31.8–47.2)
Obese	80	30.6 (23.7–38.4)
**Any contraceptive use**		
No, not currently in a sexual relationship	17^c^	5.9 (3.1–10.7)^c^
No, in a sexual relationship but not doing anything to prevent pregnancy	43	15.6 (10.8–22.2)
Yes	197	78.5 (71.4–84.2)
**Any cigarette smoking** ^f^	43	18.5 (12.8–25.9)
**Any e-cigarette use** ^g^	11^c^	4.3 (2.1–8.8)^c^
**Any alcohol use** ^h^	152	84.3 (75.9–90.2)
**Any heavy drinking**^i^	^e^	^e^
**Any binge drinking in past 30 days**^j^	31	13.1 (8.4–19.8)
**Any marijuana use** ^k^	13^c^	5.1 (2.5–10.1)^c^
**Any depressive symptoms** ^l^	29	10.2 (6.4–15.8)
**General perception of health**		
Excellent	68	25.4 (19.3–32.8)
Very good	100	38.9 (31.6–46.7)
Good	73	30.4 (23.5–38.3)
Fair	19	5.2 (2.8–9.6)

^a^ Unweighted sample size; overall unweighted sample size is 268.

^b^ Weighted percentage denominators for some measures may differ due to missing data. Father analysis weight is the product of his sampling weight, non-response weight and non-coverage.

^c^ Relative standard error (RSE) for the estimate is between 30–50%; estimates should be interpreted with caution.

^d^ Among respondents who had attended any healthcare visit since their infant’s mother was pregnant or since their infant’s birth. Respondents were asked to check all types of visits they had attended.

^e^ RSE>50% or standard error equal to zero; results not shown.

^f^ Defined as smoking any cigarettes on an average day since the baby was born.

^g^ Defined as smoking any e-cigarettes on an average day since the baby was born. E-cigarettes were defined as electronic cigarettes and other electronic nicotine vaping products (such as vape-pens, e-hookahs, hookah pens, e-cigars, e-pipes) which are battery-powered devices that use nicotine liquid rather than tobacco leaves, and produce vapor instead of smoke.

^h^ Defined as consuming any alcoholic drink in an average week since the baby was born. A drink was defined as 1 glass of wine, wine cooler, can or bottle of beer, shot of liquor, or mixed drink.

^i^ Defined as consuming 14 or more alcoholic drinks in an average week since the baby was born.

^j^ Defined as consuming 5 or more alcoholic drinks in on at least one day in the past 30 days.

^k^ Reported use of marijuana since the baby was born.

^l^ Defined as reporting “always” or”often” feeling down, depressed, or hopeless or having little interest or pleasure in doing things he usually enjoyed since the baby was born.

With regard to paternal mental and physical health, 10.2% fathers reported depressive symptoms since their infant’s birth. Most fathers reported their health status as excellent (25.4%) or very good (38.9%); the remainder reporting good or fair. Over two-thirds of fathers were overweight (39.2%) or had obesity (30.6%) based on self-reported weight and height at the time of survey completion. Most fathers reported consuming any alcoholic drink in an average week (84.3%) since their infant’s birth, with 13.1% reporting any binge drinking (5 or more drinks on one occasion) in the past 30 days. Overall, 18.5% of fathers reported smoking cigarettes on an average day at the time of survey completion. Since their infant was born, 4.3% reported e-cigarette use and 5.1% reported marijuana use. Most fathers reported they and the infant’s mother were currently doing something to prevent pregnancy (78.5%), while 15.6% reported currently being in a sexual relationship with the mother but not doing anything to prevent pregnancy, and 5.9% reported not currently being in a sexual relationship with the infants mother (Table 4).

## Discussion

This pilot surveillance effort to adapt PRAMS methodology to include fathers around the time of an infant’s birth was successful in testing two different approaches for contacting fathers while also collecting representative data for fathers’ perinatal health behaviors. Demographic characteristics and response rates were similar between the two randomized arms. In comparing survey respondents and non-respondents, we found similar sociodemographic characteristics between most subgroups, with the exception of lower response rates among fathers who were non-Hispanic Black or unmarried. By measuring the health behaviors of men during the perinatal period, the pilot PRAMS for Dads study helps bridge a research gap in understanding the health of fathers during this key timepoint in men’s lifecourse.

### Preferred approaches to reaching fathers

Historically, as prior research has shown, studies of fathers during the perinatal period experience the highest participation rate when interviews are conducted with in-person options, whether in hospital or in the home. For example, the Fragile Families and Child Wellbeing Study interviewed both mothers and fathers, recruiting in the hospital around the time of birth, with both in-person and telephone completion available; 76% of fathers completed interviews when mothers had already completed the interview. Overall response rates for fathers were higher within the hospital (70%) compared with outside the hospital (53%) due to ease of locating fathers during hospital visits [25]. The Pregnancy Risk Assessment Monitoring System-Zika Postpartum Emergency Response (PRAMS-ZPER) study, conducted in Puerto Rico during the 2017 Zika outbreak, also achieved a high response rate for fathers (77%) through the use of self-administered surveys, completed in-person in the birth hospital [9]. The Early Childhood Longitudinal Study–Birth Cohort used interviews and self-administered questionnaires during in-home visits. The study achieved a 56% response rate among resident fathers and a 37% response rate for nonresidential fathers of nine-month old infants, suggesting that reaching this latter group of fathers may be particularly challenging. Response rates from these studies add to the research showing that contacting fathers in-person may yield higher response [19].

In the current study, a unique aspect was testing two approaches to reach fathers via survey methodology. In this study, no major differences were noted between the two arms in terms of demographics, including whether parents were married or unmarried. Given the similarity of response rates between the two arms, future decision-making regarding the preferred approach for implementing a similar type of study might depend on staffing needs or resources available for reaching a representative sample of fathers. An alternative study design, such as using a hybrid approach where the two methodological arms are merged, could also be considered. For example, researchers could initially contact fathers directly and then use the indirect approach whereby mailings are addressed to mothers when attempting to reach non-respondent fathers. Nonetheless, the Indirect-to-Dads approach may be less burdensome on staff and resources as compared to the Direct-to-Dads approach, as efforts may be included along with ongoing sampling of mothers in PRAMS or similar maternal-infant health surveillance surveys. The Indirect-to-Dads approach would also need to be balanced against the theoretical burden placed on mothers as the vehicle for delivery of the survey to fathers. Additionally, telephone contact was far more labor intensive compared with the other two modes of data collection, with web-based survey completion being the least labor intensive. The lack of adequate contact information for fathers in birth certificate files and external databases made contact by telephone especially challenging. While attempting to locate and reach fathers directly could theoretically be a better approach to survey fathers with lower response rates, this approach requires more intensive use of resources to identify the father’s contact information.

Based on the PRAMS-ZPER study [9] and our findings in Georgia, earlier postnatal engagement of men with a father-focused survey may also be advantageous. The PRAMS-ZPER survey used in-hospital assessments to reach fathers shortly after their infant’s birth. This timing, combined with in-person contact, may prove beneficial for reaching more fathers overall and to garner increased interest in subsequent reporting on new fatherhood experiences that may impact father or family health. Online survey completion also holds promise for data collection, as a quarter of PRAMS for Dads respondents opted to complete surveys online. The use of a web-based option for survey completion required less staffing time, as no data entry or telephone calling was required. Future public health surveillance systems aimed at reaching new fathers can consider postnatal timing of survey and determine the most appropriate study design based on available resources, data collection mode (e.g., in-person, mailings, web, telephone), and timely access to databases with information needed for contacting fathers.

### Father health and healthcare characteristics and behaviors

Population-based estimates from PRAMS for Dads on select indicators of fathers’ mental and physical health in Georgia appear to be similar to estimates from other studies examining these characteristics in men. For example, the prevalence of postnatal depressive symptoms (10.2%) in our sample is slightly lower than the North American prevalence estimate (12.5%) reported in a meta-analysis of paternal perinatal depression studies published between 1980 and 2015 [29]. The study also highlighted that the highest prevalence of depression was observed 3–6 months after the birth of a child (13.0%), approximately the same period as PRAMS for Dad’s data collection. The prevalence of men who had obesity (30.6%) or overweight (39.2%) in our sample was also comparable to the overall prevalence of obesity for men in Georgia (32.6% obese, 38.4% overweight; BRFSS) [30].

Select health behaviors assessed in this study are also similar to those found in state-sponsored representative surveys. For example, the 2019 BRFSS survey reported 19.0% of adult men in Georgia were current tobacco smokers, and 20.0% engaged in binge drinking during the prior month [30]. We found 18.5% of fathers used cigarettes on an average day, and 13.1% engaged in binge drinking since their infant was born. While the measures of cigarette use and binge drinking were not directly comparable due to differences between our study (sampling fathers) and Georgia BRFSS (sampling all adult men) as well as differences in questions used to assess use, these findings suggest behaviors of men who recently became fathers in Georgia may be similar to those of the adult male population in Georgia.

Among respondents, 15.6% were currently sexually active with the infant’s mother but not using contraception, increasing risks of rapid repeat pregnancy. Prevention of rapid repeat pregnancy reduces the likelihood of short interpregnancy intervals (less than 18 months), which are associated with adverse maternal and child outcomes [31]. Future analyses of PRAMS data from mothers matched with PRAMS data for fathers will provide a more complete picture of contraceptive use among couples with a recent live birth in Georgia.

### Limitations

Our findings from the pilot study of fathers with a recent live birth are subject to several limitations. Most notable is the overall response rate (31.7%), with lower response rates from unmarried and non-Hispanic Black fathers; in comparison, Georgia PRAMS (mothers with a recent live birth) had a response rate of 59% in 2018 and 61% in 2019 (https://www.cdc.gov/prams/prams-data/response-rate-tables/2019-response-rate-table.html). The web-based option available for PRAMS for Dads, but unavailable for PRAMS, makes comparisons in response rates inexact. Nevertheless, our lower response rate among fathers compared with mothers is consistent with two earlier studies observing the same among parents in the newborn period [19, 32]. Relying on birth certificate data to obtain father’s contact information is another limitation as it only includes their physical (residential) address, unlike for mothers, which generally includes both their mailing address and telephone number. The limitations of and reliance on birth certificate data then meant that, during the telephone phase of the Direct-to-Dads arm, mothers were contacted to retrieve mail non-respondent fathers’ contact information, an approach that subsequently utilized mothers to reach fathers. Further, in reaching fathers via two methodological arms, there may be biases for each approach, including an overrepresentation of fathers already interested in fatherhood and health behaviors in the Direct-to-Dads arm, while the Indirect-to-Dads arm fathers may provide survey responses made to appeal to their partners, or overseen by partners. While the study enabled testing for differences in sociodemographics, a more nuanced examination of selection bias was not possible, and future research should consider the objectives of and potential biases when considering best approaches for reaching fathers. Finally, given we limited our analysis to those fathers listed on birth certificate as married or unmarried with a signed paternity acknowledgement form, it may not capture same-sex, adoptive, surrogate, those unmarried fathers unable to sign (incarcerated, deployed) or absentee fathers. Of the latter, it is important to note that 20% of infants with mothers sampled for Georgia PRAMS had no father listed on their birth certificate. Whether this indicates that the father is absent or there are other reasons for not acknowledging paternity on the birth certificate is unknown.

### Public health implications

Studies accounting for contextual factors linked to health behaviors and using representative samples of fathers can help capture the full lifecourse experiences of men who become fathers in the United States. While this study advances the approaches useful in reaching this understudied population, there are family-level considerations inherent in these efforts. In the Indirect-to-Dads approach, requiring mothers to facilitate the provision of surveys to fathers may contribute to increased maternal burdens of assuming responsibility for family health and wellbeing. In turn, one way to reduce these burdens may be to reduce bias within medical systems that may minimize expectations of fathers’ roles and responsibilities to their families, especially in the area of health care utilization [33].

The PRAMS for Dads study lies at the unique intersection of family and men’s health around the time of the birth of a new infant. This study contributes to our understanding of the important role becoming a father may play in men’s health, health behaviors, and healthcare use in this time period, and the importance fathers can play in their families’ lives. Studies such as PRAMS for Dads can help quantify men’s health behaviors and service needs, which can be used to inform recommended clinical services such as screening for alcohol use, substance use, depression, oral health, and weight management [34–38]. Targeted focus in these areas may lead to better understanding of and interventions aimed at men’s increased morbidity from chronic conditions [39], riskier behaviors and reduced life expectancy. Researchers also suggest incorporating discussion and measurement of masculinity norms into health promotion, as endorsement of these norms is linked to riskier health behaviors [40, 41]. Prioritizing and expanding studies focused on further understanding men’s health during the transition to parenthood can elucidate this time as a potential lever for change. Advancing understanding of health and wellbeing for fathers can provide valuable information used to improve programming, policy, and health for fathers and families.

## Supporting information

S1 Table PRAMS for dads pilot survey questions and references. (DOCX)

## References

[1] Monte L, Knop B. Men’s fertility & fatherhood: 2014. Washington, DC: U.S. Census Bureau; 2019.

[2] Garfield CF, Simon CD, Harrison L, Besera G, Kapaya M, Pazol K, et al. Pregnancy Risk Assessment Monitoring System for Dads: Public health surveillance of new fathers in the perinatal period. Am J Public Health. 2018;108(10):1314–5. doi: 10.2105/AJPH.2018.304664 30207766 PMC6137775

[3] Hunter T, Cattelona G. Breastfeeding initiation and duration in first-time mothers: exploring the impact of father involvement in the early post-partum period. Health Promot Perspect. 2014;4(2):132–6. doi: 10.5681/hpp.2014.017 25649998 PMC4300437

[4] Mallette JK, Futris TG, Oshri A, Brown GL. Paternal support and involvement in unmarried fragile families: Impacts on long-term maternal mental health. Fam Process. 2020;59(2):789–806. doi: 10.1111/famp.12456 31012095

[5] Martin LT, McNamara MJ, Milot AS, Halle T, Hair EC. The effects of father involvement during pregnancy on receipt of prenatal care and maternal smoking. Matern Child Health J. 2007;11(6):595–602. doi: 10.1007/s10995-007-0209-0 17557201

[6] Yargawa J, Leonardi-Bee J. Male involvement and maternal health outcomes: systematic review and meta-analysis. J Epidemiol Community Health. 2015;69(6):604–12. doi: 10.1136/jech-2014-204784 25700533 PMC4453485

[7] Sarkadi A, Kristiansson R, Oberklaid F, Bremberg S. Fathers’ involvement and children’s developmental outcomes: a systematic review of longitudinal studies. Acta Paediatr. 2008;97(2):153–8. doi: 10.1111/j.1651-2227.2007.00572.x 18052995

[8] Cabrera N, Volling B, Barr R. Fathers are parents, too! Widening the lens on parenting for children’s development. Child Development Perspectives. 2018;12(3):152–7.

[9] Salvesen von Essen B, Kortsmit K, D’Angelo D, Warner L, Smith R, Simon C, et al. Opportunities to address men’s health during the perinatal period: Lessons learned from Puerto Rico. MMWR Morb Mortal Wkly Rep. 2021;69(5152):1638–41. doi: 10.15585/mmwr.mm695152a2 33382678 PMC9191901

[10] Bronte-Tinkew J, Moore KA, Matthews G, Carrano J. Symptoms of major depression in a sample of fathers of infants: Sociodemographic correlates and links to father involvement. Journal of family issues. 2007;28(1):61–99.

[11] Price-Robertson R, Baxter J, Mathews S. Longitudinal associations between fathers’ mental health and the quality of their coparenting relationships. Clinical psychologist. 2017;21(3):215–26.

[12] Brophy S, Rees A, Knox G, Baker JS, Thomas NE. Child fitness and father’s BMI are important factors in childhood obesity: a school based cross-sectional study. PLoS One. 2012;7(5):e36597. doi: 10.1371/journal.pone.0036597 22693553 PMC3365059

[13] Allport BS, Johnson S, Aqil A, Labrique AB, Nelson T, Kc A, et al. Promoting father involvement for child and family health. Acad Pediatr. 2018;18(7):746–53. doi: 10.1016/j.acap.2018.03.011 29653255

[14] Kotelchuck M, Lu M. Father’s role in preconception health. Matern Child Health J. 2017;21(11):2025–39. doi: 10.1007/s10995-017-2370-4 28983715

[15] Schiller JS, Lucas JW, Peregoy JA. Summary health statistics for US adults: national health interview survey, 2011. Vital Health Stat 10. 2012(256):1–218. 25116400

[16] Balluz L, Easton A, Garcia D, Garvin W, Kambon M, MacDonald G, et al. Prevalence of selected risk behaviors and chronic diseases-Behavioral Risk Factor Surveillance System (BRFSS), 39 step communities, United States, 2005. Morbidity and Mortality Weekly Report (MMWR) Centers for Disease Control—Surveillance Summaries. 2008;57(SS11-October 31, 2008):1–20. 18971922

[17] Martinez GM, Daniels K, Febo-Vazquez I. Fertility of men and women aged 15–44 in the United States: National Survey of Family Growth, 2011–2015. Natl Health Stat Report. 2018(113):1–17. 30248009

[18] Ogden CL, Fakhouri TH, Carroll MD, Hales CM, Fryar CD, Li X, et al. Prevalence of obesity among adults, by household income and education—United States, 2011–2014. MMWR Morb Mortal Wkly Rep. 2017;66(50):1369–73. doi: 10.15585/mmwr.mm6650a1 29267260 PMC5751581

[19] Avenilla F, Rosenthal E, Tice P. Fathers of US children born in 2001: Findings from the Early Childhood Longitudinal Study, Birth Cohort (ECLS-B) ED TAB. NCES 2006–002. National Center for Education Statistics. 2006.

[20] Shulman HB, D’Angelo DV, Harrison L, Smith RA, Warner L. The Pregnancy Risk Assessment Monitoring System (PRAMS): Overview of design and methodology. Am J Public Health. 2018;108(10):1305–13. doi: 10.2105/AJPH.2018.304563 30138070 PMC6137777

[21] Georgia Department of Public Health, State Office of Vital Records, Revision March 2018. Paternity Acknowledgement Form. Form 3940. State Office of Vital Records, Revision March 2018.

[22] Schoppe-Sullivan SJ, Altenburger LE, Lee MA, Bower DJ, Kamp Dush CM. Who are the gatekeepers? Predictors of maternal gatekeeping. Parent Sci Pract. 2015;15(3):166–86. doi: 10.1080/15295192.2015.1053321 27366115 PMC4922533

[23] Allen S, Hawkins A. Maternal gatekeeping: Mothers’ beliefs and behaviors that inhibit greater father involvement in family work. Journal of marriage and the family. 1999:199–212.

[24] Fagan F, Cherson M. Maternal gatekeeping: The associations among faciliation, encouragement, and low-income fathers’ engagement with young children. Journal of family issues. 2017;38:633–53.

[25] Reichman N, Teitler J, I G, SS M. Fragile families: Sample and design. Children and Youth Services Review. 2001;23(4–5):303–26.

[26] Harris PA, Taylor R, Thielke R, Payne J, Gonzalez N, Conde JG. Research electronic data capture (REDCap)—a metadata-driven methodology and workflow process for providing translational research informatics support. J Biomed Inform. 2009;42(2):377–81. doi: 10.1016/j.jbi.2008.08.010 18929686 PMC2700030

[27] Benjamini Y, Hochberg Y. Controlling the false discovery rate: a practical and powerful approach to multiple testing. J R Stat Soc. 1995;57:289–300.

[28] Fleiss J, Levin B, Paik M. Statistical methods for rates and proportions (3rd ed). Hoboken, N.J.: Wiley; 2003.

[29] Cameron EE, Sedov ID, Tomfohr-Madsen LM. Prevalence of paternal depression in pregnancy and the postpartum: An updated meta-analysis. J Affect Disord. 2016;206:189–203. doi: 10.1016/j.jad.2016.07.044 27475890

[30] Centers for Disease Control and Prevention. National Center for Chronic Disease Prevention and Health Promotion, Division of Population Health. BRFSS Prevalence & Trends Data [online]. [accessed Apr 16, 2021]. URL: https://www.cdc.gov/brfss/brfssprevalence/. 2015.

[31] Conde-Agudelo A, Rosas-Bermudez A, Castano F, Norton MH. Effects of birth spacing on maternal, perinatal, infant, and child health: a systematic review of causal mechanisms. Stud Fam Plann. 2012;43(2):93–114. doi: 10.1111/j.1728-4465.2012.00308.x 23175949

[32] Carlson M, McLanahan S. Fathers in Fragile Families. The role of the father in child development. 2010;5:241–69.

[33] Frascarolo F, Feinberg M, Sznitman G, Favez N. Professional gatekeeping toward fathers: A powerful influence on family and child development. Perspectives in Infant Mental Health. 2016:1–7.

[34] Chou R, Dana T, Blazina I, Grusing S, Fu R, Bougatsos C. Interventions for unhealthy drug use-Supplemental Report: A systematic review for the US Preventive Services Task Force. U.S. Preventive Services Task Force Evidence Syntheses, formerly Systematic Evidence Reviews. Rockville (MD)2020.

[35] Siu AL, Force USPST, Bibbins-Domingo K, Grossman DC, Baumann LC, Davidson KW, et al. Screening for depression in adults: US Preventive Services Task Force Recommendation Statement. JAMA. 2016;315(4):380–7. doi: 10.1001/jama.2015.18392 26813211

[36] O’Connor EA, Perdue LA, Senger CA, Rushkin M, Patnode CD, Bean SI, et al. Screening and behavioral counseling interventions to reduce unhealthy alcohol use in adolescents and adults: Updated evidence report and systematic review for the US Preventive Services Task Force. JAMA. 2018;320(18):1910–28. doi: 10.1001/jama.2018.12086 30422198 PMC13585200

[37] LeBlanc ES, Patnode CD, Webber EM, Redmond N, Rushkin M, O’Connor EA. Behavioral and pharmacotherapy weight loss interventions to prevent obesity-related morbidity and mortality in adults: Updated evidence report and systematic review for the US Preventive Services Task Force. JAMA. 2018;320(11):1172–91. doi: 10.1001/jama.2018.7777 30326501 PMC13151892

[38] Lipsky MS, Su S, Crespo CJ, Hung M. Men and oral health: A review of sex and gender differences. Am J Mens Health. 2021;15(3):15579883211016361. doi: 10.1177/15579883211016361 33993787 PMC8127762

[39] Baker P, Shand T. Men’s health: time for a new approach to policy and practice? J Glob Health. 2017;7(1):010306. doi: 10.7189/jogh.07.010306 28400949 PMC5344012

[40] Houle J, Meunier S, Coulombe S, Tremblay G, Gaboury I, De Montigny F, et al. Masculinity ideology among male workers and its relationship to self-reported health behaviors. International Journal of Men’s Health. 2015;14(2):163–82.

[41] Mahalik JR, Burns SM, Syzdek M. Masculinity and perceived normative health behaviors as predictors of men’s health behaviors. Soc Sci Med. 2007;64(11):2201–9. doi: 10.1016/j.socscimed.2007.02.035 17383784

