# Supplementary material for: Pregnancy Risk Assessment Monitoring System for Dads: A piloted randomized trial of public health surveillance of recent fathers’ behaviors before and after infant birth
Source: PLoS One. 2022 Jan 21;17(1):e0262366. doi: 10.1371/journal.pone.0262366 (PMC8782358; doi:10.1371/journal.pone.0262366)
Supplement: S1 Table — (DOCX) [file pone.0262366.s001.docx]

**S1 Table. PRAMS for dads pilot survey questions and references.**

| Questions | Topic | Source |
| --- | --- | --- |
| 1 | Date of birth | PRAMS phase 8 core |
| 2-4 | Height and weight | PRAMS phase 8 core |
| 5-8 | Health care use -prenatal | PRAMS phase 8 core for Q5 and Q6, modified for Q7 and Q8 |
| 9 | Residency with baby’s mother – prenatal |  |
| 10 | Relationship status – prenatal | PRAMS phase 8 standard |
| 11 | Pregnancy intendedness | PRAMS phase 8 core |
| 12-14 | Birth control use - prenatal | PRAMS phase 8 core |
| 15-17 | Cigarette use | PRAMS phase 8 core |
| 18-20 | E-cigarette use | PRAMS phase 8 core |
| 21-24 | Alcohol use | PRAMS phase 8 core |
| 25-26 | Substance use | PRAMS phase 8 standard and drug supplement |
| 27 | Baby birthdate | PRAMS phase 8 core |
| 28-30 | Health care use - postnatal | PRAMS phase 8 core for Q28 and Q29, modified for Q30 |
| 31 | Residency with baby’s mother – postnatal |  |
| 32-33 | Relationship status - postnatal | PRAMS phase 8 standard (Q32), Fragile Families (Q33) |
| 34 | Baby living | PRAMS phase 8 core |
| 35-39 | Breastfeeding | PRAMS phase 8 core (Q35-Q37), PRAMS phase 8 standard, modified (Q38 and Q39) |
| 40-42 | Safe sleep | PRAMS phase 8 core |
| 43 | Residency with baby | Fragile Families |
| 44-45 | Non-residential fathering | 2011-2013 NSFG (Male), Section G: Fathering |
| 46-47 | Attitude about fatherhood | Fragile Families |
| 48-51 | Father-infant involvement | PRAMS state-submitted CT75 (Q48), Fragile Families (Q49), Parenting Sense of Competence (PSOC; Q50,51) |
| 52-53 | Health insurance | PRAMS (options from Georgia Phase 8) |
| 53 | Safety | PRAMS phase 8 standard |
| 55-56 | Incarceration and parole | Fragile Families |
| 57-58 | Birth control use - postnatal | PRAMS phase 8 core |
| 59-61 | Self-reported physical and mental health | PRAMS phase 8 standard (Q59), PHQ-2 (Q60-61) |
| 62-64 | Other children |  |
| 65-69 | Employment and work leave | PRAMS phase 8 standard |
| 70-71 | Income | PRAMS phase 8 core |
| 72 | Today’s date | PRAMS phase 8 core |
|  | Contacting and additional comments |  |
